# Comment on: Proposal for a new diagnostic classification of photodistributed Stevens–Johnson syndrome and toxic epidermal necrolysis

**DOI:** 10.1186/s40001-024-01652-7

**Published:** 2024-01-29

**Authors:** Bukiwe N. Thwala, Nadine Teixeira, Eddy Zitha, Aneliswa Mpungose, Thuraya Isaacs, Jonathan G. Peter, Rannakoe J. Lehloenya

**Affiliations:** 1 Division of Allergy and Clinical Immunology, Department of Medicine, Groote Schuur Hospital and University of Cape Town, Cape Town, South Africa; 2 Division of Dermatology, Department of Medicine, Groote Schuur Hospital and University of Cape Town, Cape Town, South Africa; 3 Allergy and Immunology Unit, University of Cape Town Lung Institute, Cape Town, South Africa; 4 Combined Drug Allergy Clinic and Groote Schuur Hospital, Cape Town, South Africa

## Abstract

Stevens–Johnson syndrome (SJS), toxic epidermal necrolysis (TEN), and SJS/TEN overlap (SJS/TEN), collectively referred to SJS/TEN, form a spectrum of severe life-threatening adverse drug reactions whose pathomechanism is not fully understood. The article "Photodistributed Stevens–Johnson Syndrome and Toxic Epidermal Necrolysis: A Systematic Review and Proposal for a New Diagnostic Classification" by McKinley et. al., discusses a distinct distribution of epidermal necrosis in SJS/TEN, attributable to preceding exposure to ultraviolet radiation (UVR), and relative sparing of photo-protected areas. After reviewing numerous cases within the Immune-mediated Adverse drug Reactions in African HIV endemic setting Register and Biorepository (IMARI-SA) at the University of Cape Town with a similar clinical pattern as those published by McKinley et. al., we propose that the relative sparing of some areas giving an impression of photo-distribution is due to localised increase in skin pressure that reduces the blood supply in that area below a critical threshold. A dip in blood supply below this critical threshold quantitively limited T lymphocytes and cytokines that drive SJS/TEN to reach and damage the skin.

**The Editor,**

We read with interest the article by McKinley, Allen, and Michels titled "Photodistributed Stevens–Johnson Syndrome and Toxic Epidermal Necrolysis: A Systematic Review and Proposal for a New Diagnostic Classification". The authors reviewed a total of 13 SJS/TEN cases that reported preceding and temporally related ultraviolet radiation (UVR) exposure to support their hypothesis that UVR was responsible for the distinct distribution of epidermal necrosis in these cases [1]. We commend the authors for their thorough and detailed review that makes a valuable contribution to the SJS/TEN literature while stimulating a discussion. Nonetheless, we would like to highlight some limitations of the article, which, if addressed, would strengthen its conclusions. Additionally, we offer an alternative explanation for the clinical pattern described in some of the reported cases.

In a study proposing a new subtype of SJS/TEN and its classification, the onus lies with the authors to as much as possible to only include true cases. Use of these validation scores to include only definite (possibly probable) cases would ensure that they meet the generally acceptable case definition of SJS/TEN. This would allow the authors to describe the differences more precisely with SJS/TEN as generally accepted. The RegiSCAR and J-SCAR validation criteria are the two most widely used validation scores [2, 3]. Closer scrutiny of each case and how SJS/TEN was defined, rather than basing inclusion on the authors’ opinion formally designating the case as SJS/TEN, would further strengthen the article. Similarly, it is our view that the inclusion of five cases with a final ALDEN score of 2–3 (possible) falls short of internationally acceptable standards. Although this does not impact case definition in the current article, it has a bearing on the case inclusion criteria and ascribing drug causality in the proposed future studies to validate the author’s findings and classification.

Photo-induced reactions generally start in photoexposed areas. In the case of immune-mediated forms, photoallergic reactions are a prime example, and the rash subsequently progresses to photoprotected areas [4]. McKinley et al. specifically addressed this by excluding cases with a sharp cutoff typical of phototoxic reactions. However, they described SJS/TEN lesions beyond sun-exposed areas at first presentation, including mucosal and palmoplantar involvement in 12/12 and 5/6 cases, respectively [1]. Detailing the sequence in which the photoexposed and protected areas were initially affected would be helpful, otherwise this brings to question the veracity of attributing causality to UVR as an explanation for this clinical pattern.

We propose that some of the areas that were relatively spared represent areas of increased local pressure on the skin rather than photo-induced or aggravated pattern. We have often seen the pattern illustrated in the images published by McKinley et al. in our SJS/TEN patients. We usually interpreted these as areas of relative sparing due to pressure from clothes and/or from prolonged anatomical positions that patients assume during the early stages of an evolving SJS/TEN. We reviewed SJS/TEN cases in our Immune Mediated Adverse Reactions in Africa (IMARI-Africa) database, a multinational, multicentre prospective registry, and biorepository of severe adverse drug reactions [5]. We found numerous cases with a clinical pattern similar to the clinical images published in the article, as illustrated in Figs. 1 and 2**.** IMARI-Africa is approved by the University of Cape Town Human Research Ethics Committee, and the patients shown in these pictures gave written consent for publication of their non-identifying images in a scientific journal.

**Fig. 1:**
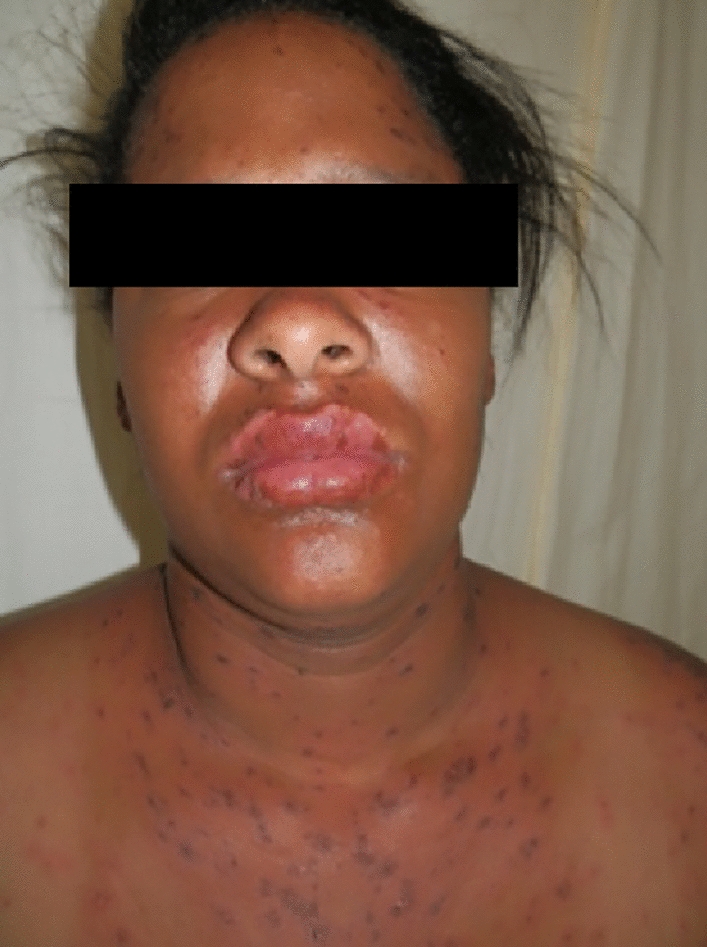
Rash distribution similar to that published by McKinley et al. Relative sparing of areas covered by a brassiere

**Fig. 2:**
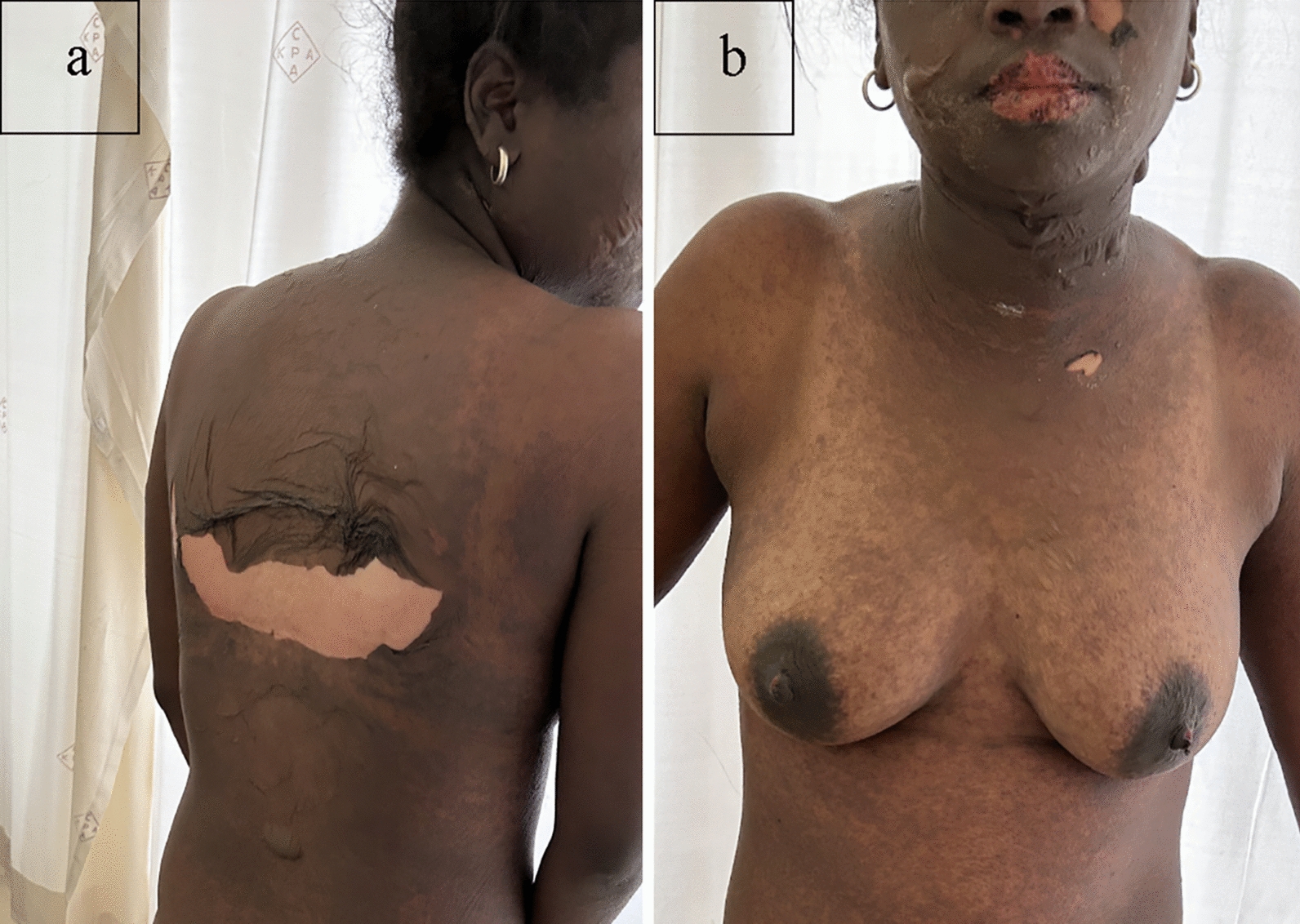
Rash distribution similar to that published by McKinley et al. Relative sparing of areas covered by a brassiere

We hypothesize that areas of sustained pressure on the skin reduce the blood supply below a critical threshold that prevents pathogenic T lymphocytes and cytokines from damaging the skin. Once the pressure is relieved, the disease progresses to involve these areas dependent on the circulating quantities of these cells and cytokines at that time point. We further hypothesize that the critical threshold and duration of vascular impairment to the skin’s blood supply is well above that required for skin necrosis under normal circumstances.

We believe that our insights offer another perspective on the clinical patterns observed by McKinley et al. in SJS/TEN. We hope our contribution stimulates further dialogue and encourages continued exploration into the intricate mechanisms underlying this potentially life-threatening reaction. Furthermore, we aim to systematically review all our cases in the near future to determine the veracity of our hypothesis versus that of McKinley et al.

## Data Availability

Not applicable.
